# Editorial: Social Convergence in Times of Spatial Distancing: The Role of Music During the COVID-19 Pandemic

**DOI:** 10.3389/fpsyg.2022.910101

**Published:** 2022-05-25

**Authors:** Niels Chr. Hansen, Melanie Wald-Fuhrmann, Jane W. Davidson

**Affiliations:** ^1^Aarhus Institute of Advanced Studies, Aarhus University, Aarhus, Denmark; ^2^Center for Music in the Brain, Department of Clinical Medicine, Aarhus University and the Royal Academy of Music, Aarhus, Denmark; ^3^Department of Music, Max Planck Institute for Empirical Aesthetics, Frankfurt am Main, Germany; ^4^Max Planck NYU Center for Language, Music and Emotion (CLaME), Frankfurt am Main, Germany; ^5^Melbourne Conservatorium of Music, Faculty of Fine Arts and Music, University of Melbourne, Melbourne, VIC, Australia

**Keywords:** music, COVID-19, coping, wellbeing, creativity, music education, music therapy

## Introduction

The novel coronavirus pandemic and the lockdown measures introduced to contain it had severe consequences for individuals, societies, the economy, and public life at large all around the globe (Clemente-Suárez et al., 2020). It also affected the ways that people engage with music (Wald-Fuhrmann, in press; Hansen, in press A). Long before concerts were canceled and music venues were shut down, creative outpourings from professional musicians and skilled amateurs appeared, and new patterns of music consumption started spreading as “virally” as COVID itself throughout the world (Fraser et al.; Hansen et al.). In fact, Google search trends for “music AND corona” suggest that musical responses to the surging outbreak took off already in February 2020 thus preceding the pandemic declaration by the World Health Organization on 11th March by several weeks (Hansen, in press B). The abundant and diverse Frontiers Research Topic presented here arose out of this context.

Pandemic musical responses were multifarious, though further work is required to map and fully understand them (cf. Wald-Fuhrmann, in press; Hansen, in press A). A new trend in music creation emerged, with reworkings of existing music or new compositions on the theme of COVID and/or the lockdown itself.^1^ This work often included fun and thought-provoking videos that proliferated on social media platforms like YouTube and TikTok under hashtags such as #coronasongs, #quarantunes, and #songsofcomfort (Jones, 2020). Simultaneously, leading symphony orchestras, opera houses, bands, and concert venues embraced digital formats for sharing recordings and live streams to rejuvenate their public appearance and maintain their core audience (Bergman, 2021). Celebrity musicians invited fans into their private homes, performing in casual attire for televised virtual charity events like “One World: Together at Home” (18 April 2020) and “One Love Asia Concert” (27 May 2020; McIntosh, 2021). The general public engaged in daily rituals such as clapping for healthcare workers and singing from balconies (Calvo and Bejarano, 2020; Cabedo-Mas et al.).

Choirs and musical ensembles whose regular rehearsals were disrupted found new ways of making music together online–either synchronously in real-time (Daffern et al.; Morgan-Ellis) or asynchronously producing the characteristic splitscreen videos that became a hallmark of the “coronamusic” phenomenon (Hansen et al.; Hansen, in press A). All forms of music teaching from the primary classroom (Parkes et al.) and extra-curricular ensembles (Levstek et al.) to graduate-level classical instrumental studios (de Bruin) moved online, sometimes with institutions commissioning home delivery services to facilitate student access to musical instruments (Davidson, 2021). As music therapists were forced to swiftly adapt their practice toward telehealth approaches, posing considerable technological challenges (Agres et al.; Cole et al.; Dowson et al.), music was also used in hospitals and intensive-care units to support COVID-19 patients feel emotionally connected to their loved ones despite physical distancing requirements (Howlin and Hansen, in press). Many musical initiatives were closely covered by the media (Hansen et al.) as general consumption of news increased during lockdown (Fink et al., 2021). Some of these reports were, however, biased to favor certain narratives (Deaville and Lemire) and sometimes gave an overly optimistic view of music's alleged healing powers. This latter positivity bias has been proposed to constitute a potential coping strategy to promote resilience and social cohesion during challenging times (Hansen, in press A).

### The MUSICOVID Research Network

Concerted, cross-disciplinary, critically rigorous, and culturally inclusive research efforts were required to attain an evidence-based understanding of the psychological mechanisms underlying these widespread cultural practices and innovations. To this end, Melanie Wald-Fuhrmann and Niels Chr. Hansen founded the MUSICOVID research network which was formally inaugurated during two virtual gatherings on 19th May 2020. Here, 23 talks were given to share initial research plans and preliminary findings. Because the call for contributions had been distributed widely on listservs and social media channels representing music psychology/cognition, music theory, auditory science, music therapy, ethnomusicology, systematic musicology, and musicology at large, participation was diverse in terms of scientific disciplines and geographical regions. The establishment of the network was motivated by a growing concern that music researchers would produce underpowered and redundant studies tackling overlapping basic research questions about the quantity, quality, and effects of musical engagement during home confinement. By joining forces–or at least disseminating theoretical perspectives and empirical research designs early on–resources could ideally be spent more wisely and hopefully enable swift advancements toward more nuanced and sophisticated hypothesis formulation.

At the time of writing this editorial, about 2 years later, the MUSICOVID network comprises 419 members from more than 250 universities and other organizations based in 49 countries on all six inhabited continents. Around the time of the inauguration, we guest editors realized that the Frontiers Research Topic model would constitute a highly suitable channel for presenting findings from a large number of MUSICOVID members under a single topical umbrella. Indeed, many contributions to the current Research Topic feature network members and–in some cases–international research collaborations formed under the auspices of MUSICOVID.

## The Pandemic Music Research Landscape

The pandemic music research efforts featured here covered a data collection timespan from February 2020 to January 2021^2^ and, importantly, were characterized by diversity along multiple dimensions. Below, we will detail and evaluate the resulting convergences of topics, methodologies, disciplines, institutions, timelines, and geographic origins.

### Convergences of Topics, Methods, and Affiliations

**Figure 1:**
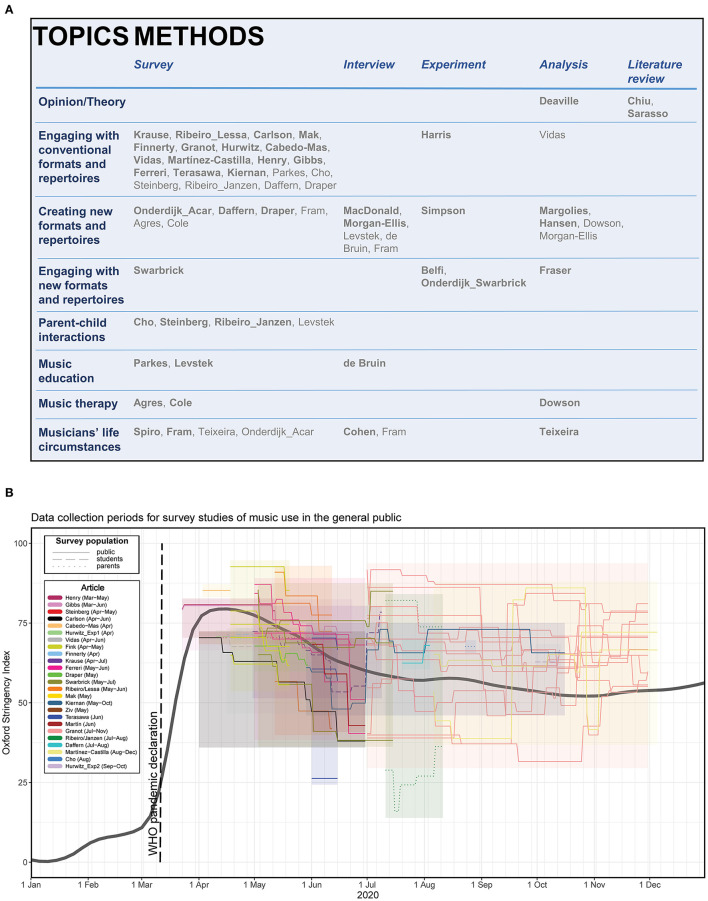
**(A)** Overview of the methods applied to each of the overall topics within the current Research Topic. For articles pertaining to multiple topics and/or employing multiple methods, primary topics and methods are indicated in bold. **(B)** Timeline depicting Oxford Stringency Index (Hale et al., 2021) in surveyed countries during data collection times for questionnaire-based studies of music use in the general public conducted during the pandemic lockdowns of 2020. Values for each survey are depicted in a unique color with each line representing the lockdown stringency over time for a single country. Shaded areas cover the data collection period and range of lockdown stringency within a given survey. Line type indicates the type of survey population (i.e., primarily students, parents, or members of the general public). For completeness, in addition to the 22 studies from this Research Topic meeting the criteria, a further three survey studies published elsewhere have been included (Fink et al., 2021; Martín et al., 2021; Ziv and Hollander-Shabtai, 2021). Studies surveying music use in therapeutic and educational contexts as well as those only involving professional musicians are not included.

Figure 1A depicts the combination of topics and methods categories employed in the 44 featured articles. For the purpose of creating this figure, the content of the Research Topic was subjected to reflexive thematic analysis whereby each article was assigned to categories along the dimensions of topics, methods, disciplines, and first author's primary affiliation. Category sets were arrived at through an inductive approach. Authors' country and continent of residence and the number of authors, institutions, and countries represented by each article were also identified. The first author of this editorial performed these categorizations which were subsequently checked and confirmed by the two remaining authors. A complete spreadsheet is available *via* the Open Science Framework (https://osf.io/kush9/).

Our thematic analysis revealed eight overall Research Topic categories: Theory/Opinion (represented by three articles), Engaging with conventional formats and repertoires (22), Creating new formats and repertoires (14), Engaging with new formats and repertoires (4), Parent-child interactions (4), Music education (3), Music therapy (3), and Musicians' life circumstances (5). Additionally, five overall methodological categories were identified: Surveys (29), Experiments (4), Interviews (5), Analyses (8), and Literature reviews (2). Experiments were defined as intervention studies–in person or virtually–where an independent variable was systematically manipulated. Analyses spanned from Music Information Retrieval methods applied to audio data (Vidas et al.), *via* fieldwork as participant-observer (Morgan-Ellis), to analysis of websites, news, music videos, or other public sources (Deaville and Lemire; Fraser et al.; Hansen et al.; Margolies and Strub; Teixeira et al.). While multiple Research Topics and methods could be associated with any article, a single primary topic and/or method was always assigned.

Engagement with conventional formats and repertoires was by far the most popular topic under study, but was closely succeeded by the creation of new formats and repertoires, the engagement with these, and musicians' disrupted life circumstances. In particular, pandemic-related changes in musical engagement and the impact of novel forms of listening to and making music attracted many researchers' attention. This most likely reflects the fact that music psychology (30 articles) was the most prominently represented discipline with smaller numbers for musicology/sociology (9), music therapy (3), and music education (2). Yet, far from all authors were affiliated with psychology departments. Rather, primary affiliations listed for first authors included departments of psychology/social science/health sciences (17 first authors), musicology/music (12), conservatories/schools of music (5), neuroscience (3), music technology (3), education/pedagogy (2), history (1), and information/media science (1). This alludes to substantial degrees of interdisciplinarity.

### Convergences of Disciplines, Collaborations, and Geographical Regions

Disciplinary convergence was similarly reflected in the inclusion of the three parent journals *Frontiers in Psychology* (42 articles), *Frontiers in Neuroscience* (1), and *Frontiers in Sociology* (1), represented by the Specialty Sections Cultural Psychology (16), Performance Science (12), Auditory Cognitive Neuroscience (7), Emotion Science (7), Cognition (1), and Work, Employment and Organization (1).

Typically, a team of authors co-wrote articles (range: 1−24; median: 4; mean: 4.50), and multiple institutions were most often listed amongst each team's affiliations (range: 1−14; median: 2; mean: 2.75). Although suitable comparison data from pre-pandemic times can be hard to obtain, a working hypothesis would be that music researchers pursued opportunities for multi-author and cross-institutional collaborations during lockdown, as has been observed for other scientific areas (Lee and Haupt, 2021). Indeed, a previous bibliometric analysis of three leading music psychology journals during 1973–2017 found annual means of author counts per article ranging from 1.00 (1973) to 2.72 (2012; Anglada-Tort and Sanfilippo, 2019). In light of these observations, the degree of collaboration as well as the disciplinary and institutional diversity exhibited by this particular Research Topic arguably exceed those of many others.

Given what was feasible online during lockdown, survey studies were the most prominently selected methodology with very few true experiments conducted (Figure 1A). Yet, technological solutions were adopted to interview research participants *via* Zoom (de Bruin; Cohen and Ginsborg; Fram et al.; MacDonald et al.; Morgan-Ellis) and conduct experiments virtually during lockdown (Harris and Cross; Onderdijk, Swarbrick et al.; Simpson et al.) or physically prior to lockdown (Belfi). We expect that as more sophisticated tools become widely available, music researchers will become increasingly better equipped to collect not only survey data but also valid experimental and interview data online (Honing and Ladinig, 2008; Eerola et al., 2021; Anglada-Tort et al., 2022). The variety of methods by which the creation and consumption of new virtual formats and repertoires were already investigated in this Research Topic paves a useful pathway for future researchers interested in other pertinent music-related topics. By enabling converging evidence and, in some cases, causal inference rather than merely detecting correlational associations, these methodological developments may enhance scientific quality and responsiveness during future pandemics, conflicts, or natural disasters.

Musical development, wellbeing, education, therapy, sonic arts, and cultural studies–although represented by a number of articles here–were studied by researchers within their respective fields. Some of this work was indeed published in more specialized journals. See, e.g., the special issues of *International Journal of Community Music* (Clift, 2021), of *Rock Music Studies* (Burns and Kitts, 2021), of *Journal of Sonic Studies* on “Sound at Home” (Abildgaard et al., 2021a,b), of *Journal of Music, Health, and Wellbeing* on “Musicking through COVID-19: Challenges, Adaptations, and New Practices” (Williams et al., 2021), and potentially many others. Review articles summarizing and critically synthesizing the growing literature on music during the COVID-19 pandemic are steadily emerging (Howlin and Hansen, in press; Hansen, in press A). Howlin and Hansen (in press), for example, appraise publications on music use in the general public as well as in music-therapeutic practice.

The global reach of the MUSICOVID network enabled greater participation from countries and geographical regions that have traditionally been under-represented both at academic conferences and in international publications–see, e.g., the bibliometric analysis for music psychology by Anglada-Tort and Sanfilippo (2019) where countries like USA, UK, Finland, and Australia dominated. To some extent, the current Research Topic reproduced such biases with strong representation of articles featuring authors from USA (12), UK (12), Australia (10), Canada (5), Norway (5), Spain (4), Belgium (3), Israel (3), Finland (2), Germany (2), Italy (2), Japan (2), Netherlands (2), and France (1). However, the list of remaining represented countries also features contributions beyond the 20 top-represented countries in Anglada-Tort and Sanfilippo's (2019) analysis. These include: Brazil (4), Luxembourg (2), Argentina (1), Chile (1), China (1), Colombia (1), Denmark (1), Ireland (1), Mexico (1), Portugal (1), and Singapore (1). The world map of article views maintained by Frontiers further suggests attraction of a relatively global audience, engaging many readers from the Global South.^3^ We believe the geographical diversity of the Research Topic was much facilitated by a temporary fee waiver for COVID-19-related research introduced by Frontiers in light of the exceptional circumstances. We hope the multi-dimensional convergences demonstrated here bode future progress toward decolonization and elimination of structural inequalities in scientific publishing (Istratii and Demeter, 2020)–also in music research.

## Overview of Contributions

As previously mentioned, the 44 articles contained in this Research Topic were grouped into eight primary topic categories, which we will now proceed to summarize.

### Theory/Opinion

Although basic empiricism was the main approach taken in the studies collected here, a small group of authors contributed theoretical considerations of historical and contemporary sources of musical activities during pandemics. Chiu's comparison of current musical responses with similar responses to the plague in sixteenth-century Milan offered a historically grounded framework for understanding how individuals and communities use music to fulfill emotional, social and self-relevant needs. This research topic reappeared in the majority of psychology-oriented papers. Based on a review of existing literature, Sarasso et al. pointed to the critical role of collectively shared aesthetic experiences of music, together with some ideas on the potentially adaptive function of music. In their analysis of the rapidly emerging Western media narratives, Deaville and Lemire could identify underlying cultural biases, offering vastly divergent accounts of soundscapes and collective musical responses in China, Iran, and Italy.

### Engaging With Conventional Formats and Repertoires

Across the pandemic, media reports often highlighted musical responses to COVID-19 and social-distancing policies. Changes in musical behaviors and how they might help individuals and groups fight stress, loneliness, and anxiety played a key role. Accordingly, many studies tackled these issues with online surveys. Some were multi-country studies (Carlson et al., Ferreri et al., Granot et al., Terasawa et al.), whereas others focussed on single countries (Australia: Kiernan et al.; Brazil: Ribeiro, Janzen et al.; Spain: Cabedo-Mas et al., Martínez-Castilla et al.; UK: Gibbs and Egermann, Henry et al., Mak et al.). Another sub-group examined student populations in Australia (Krause et al., Vidas et al.), Canada (Finnerty et al.), and the USA (Hurwitz and Krumhansl).

The results regarding the degree and type of changes in musical engagement are somewhat inconsistent. While Mak et al. and Hurwitz and Krumhansl did not find self-reported increases in musical engagement (for UK and US samples), other studies reported higher levels of daily musical engagement or an increased importance of music (Cabedo-Mas et al., Carlson et al., Ferreri et al., Ribeiro, Janzen et al.). Quantitative reports of decreased audio streaming (e.g., Spotify) accompanied by increased video streaming (e.g., YouTube) of music during lockdown (Sim et al., 2020; see also Carlson et al.) point toward a possibly accelerated uptake of multimodal media for music consumption and potentially inaccurate introspections about actual use (Hansen, in press B).

A number of studies compared musical activities with other domestic activities. Here, music was not only one of the most frequently performed activities but also repeatedly found to be as effective or even more effective than exercise, sleep, and changing location (see also Vidas et al.; Finnerty et al.; Cabedo-Mas et al.; Granot et al.). Interestingly, music seemed more beneficial than watching TV. Although both were equally frequent, watching films and TV did not make it into the group of activities deemed most effective for fighting stress and regulating mood (Kiernan et al.) or was even negatively related to life satisfaction (Krause et al.). However, the respective surveys did not always differentiate between watching films and series on the one hand (i.e., as a leisure activity and for distraction) and watching news on the other.

Some researchers also examined which specific pieces or types of music were perceived to be particularly important for individual coping. A particular role was demonstrated for negatively valenced music (Hurwitz and Krumhansl; Vidas et al.), nostalgic music (Ferreri et al.; Gibbs and Egermann) but also for happy and novel music. Other studies not in this Research Topic found that corona-themed repertoires were especially predictive of successful socio-emotional coping with music (Fink et al., 2021).

Although different numbers of concepts were employed to address potential benefits of musical engagement, a relatively coherent picture emerged. In line with a large body of earlier research on emotional and wellbeing effects of musical activities (Hanser, 2010), engagement in the context of COVID-19 was found to serve as a strategy for mood management (i.e., decreasing negative emotions and increasing positive ones, e.g., *via* venting, diversion, or enjoyment), but also to reduce stress, to have positive physical effects, such as relaxation or activation. In line with studies on music's potential role as a social surrogate (Schäfer et al., 2020), it also helped people feel less lonely (Cabedo-Mas et al.; Granot et al.; Ribeiro, Janzen et al., Vidas et al.). In addition to relatively few influencing sociodemographic, personal, and contextual characteristics, the individual importance of music (Martínez-Castilla et al.) or the sensitivity to musical reward (Ferreri et al.) were found to be critical factors.

There is, however, no guarantee that the available survey studies provide a complete account of all aspects of pandemic coping with music. For example, no studies were conducted sufficiently early to capture the time around the pandemic declaration and the initial upsurge in lockdown restrictions (Figure 1B) even though online search trends suggest public interest in coronamusic peaked during this time (Hansen, in press B). Different sampling periods and countries were, moreover, characterized by vastly divergent contextual circumstances. Before 1st July 2020 (Henry et al.; Gibbs and Egermann; Carlson et al.; Cabedo-Mas et al.; Hurwitz and Krumhansl's Exp. 1; Vidas et al.; Finnerty et al.; Krause et al.; Ferreri et al.; Ribeiro, Lessa et al.; Mak et al.; Terasawa et al.), restrictions were stringent but also eased a bit as the northern-hemisphere spring and summer progressed. After 1st July 2020 (Granot et al.; Martínez-Castilla et al.; Hurwitz and Krumhansl's Exp. 2), conversely, restrictions to some extent stagnated with notable individual differences between the surveyed countries. The studies by Granot et al. and Martínez-Castilla et al., for example, surveyed populations that were both far above and far below the world average in lockdown stringency.

### Creating New Formats and Repertoires

In addition to attempts to cope with existing music, many people–including laypersons and music professionals–created new forms and repertoires to process pandemic life, express their thoughts and feelings, and find ways to make music together despite social-distancing regimes. Providing a valuable source for future musicological and other analyses of these direct responses, Hansen et al. collected and annotated a representative corpus of coronamusic videos and news reports with the help of crowdsourcing from a global audience. Positive emotions like happiness, humor, togetherness, and being moved seemed especially prominent (cf. Hansen, in press A). In turn, Margolies and Strub looked at Facebook and YouTube user responses to COVID-specific Mexican música huasteca and improvised verses framed as community service acts promoting collective resilience.

How exactly music ensembles such as choirs, orchestras, or dance classes moved their activities online, was examined by several groups of researchers (Daffern et al.; Draper and Dingle; MacDonald et al.; Morgan-Ellis; Onderdijk et al.). Communities studied were from the UK, Belgium, The Netherlands, Australia, and USA. Typically, researchers were interested in which online platforms and solutions (e.g., live interaction vs. asynchronous multi-track performances) ensembles used and how they experienced their online interactions. Although frustration with the technical limitations and the lack of physical co-presence was a frequently raised concern, participants appreciated the possibility to develop their artistry and uphold community, and many experienced some wellbeing benefits. That online group music making can have positive wellbeing effects was also corroborated by an experiment comparing effects on stress, mood, and connectedness of individual and group-based online chanting sessions (Simpson et al.).

### Engaging With New Formats and Repertoires

Virtual concerts, i.e., performances streamed live or on demand *via* the internet, but also other forms of interacting with music online, were not only a signature activity during the pandemic but also a particularly interesting novel research area. Three studies examined audiences' experience of virtual concerts, either in comparison with live performances (Belfi et al.) or regarding the effects of various characteristics of virtual concerts (Onderdijk, Swarbrick et al.; Swarbrick et al.). Given that streamed concerts lack actual physical co-presence, findings regarding if and how virtual concerts can afford feelings of physical and social presence might be particularly relevant for future artistic development using these formats. Here, livestreams exhibited an advantage over on-demand streams (Onderdijk, Swarbrick et al.); giving audiences the opportunity to interact *via* a video-conferencing platform enhanced social presence relative to a non-interactive stream, and virtual-reality headsets induced feelings of physical presence and connectedness with the artist. Another, asynchronous form of virtual interaction, namely YouTube user commenting, was found to be potentially beneficial for social bonding and bridging across intercultural divides (Fraser et al.).

### Parent-Child Interactions

Music in the homes of young children overarchingly came out as an effective tool in building relationships and regulating mood during periods of uncertainty and change (Cho and Steinberg Ilari et al. in USA and Canada; Ribeiro, Janzen et al. in Brazil). Steinberg et al. demonstrated that parents engaged more frequently in musical activities with their children during lockdown than previously and also used music for their own and their child's emotional regulation. Cho and Ilari found that strategically selected recorded music could maintain or reinforce children's positive mood, but soothing and calming playlists were largely unsuccessful in shifting negative moods. Ribeiro, Janzen et al. also found an increase in music activity, with parents spending far more time with their children indoors, and self-reporting dedicating more hours to singing/playing and listening to music and to watching music videos. While caregivers overall experienced low wellbeing and high stress, those with better mental health and higher education levels made significantly more music with their children. Knowing that young children generally benefit from music for mood regulation (Saarikallio, 2009), these differences in use of music may have further increased the sociodemographic disparities in psychological coping during the pandemic (Fancourt et al., 2021).

### Music Education

Research in music education revealed that the mental health of US teachers deteriorated during extended periods of lockdown and that unplanned changes in mode of instruction during the semester increased psychological pressure (Parkes et al.). Levstek et al. explored changes in young people's lived experiences of virtual group music sessions during the first and second national lockdowns in the UK, finding that these new formats could assist in emotion management and maintenance of social belonging and musical identities. In Australia, interviews with experienced instrumental music educators revealed that teaching became more student-centered with more energy being spent on building emphatic connections and communicating in a more dialogic fashion, and with the teacher in a more guiding/coaching role to encourage student ownership and empowerment (de Bruin). While these changes were mostly framed as successful, it remains unclear how such approaches worked for less intrinsically motivated and parentally supported students and for new students who did not have a prior history of “in-person” experiences with the relevant educator.

### Music Therapy

Music therapy research explored how therapists adapted their practice, especially their use of technology, during the pandemic. Agres et al. surveyed music therapists from North America, Europe, and Asia/Oceania to reveal that use of technology for telehealth was in line with pre-pandemic increasing trends. Overall, technology was used more, valued higher, and regarded with greater optimism by practitioners in North America and Asia/Oceania who, compared to their European colleagues, also had substantially more training and experience in the area. The authors argued that more specialized training and exposure to software tailored toward music therapists' specific needs is required. With data primarily from the USA and Canada, Cole et al., found that Neurological Music Therapy can be delivered with telehealth methods. In the UK, Dowson et al. analyzed 50 online music activities to document, categorize and describe current opportunities for digital music making involving people with dementia. This enabled an assessment of barriers and facilitators to delivery and accessibility. Among other findings, online sessions enabled geographically separate groups and individuals to spend time together.

### Musicians' Life Circumstances

Research in the UK revealed that for musicians, substantial decreases in work and income were associated with lower wellbeing, and with higher depression and loneliness scores (Spiro et al.). In Brazil, opportunities at the federal, state and municipal level, as well as from the third sector, showed massive decreases in income for more than two thirds of musicians as important opportunities for performing and earning money disappeared (Teixeira et al.). In the UK, while interviewees mourned the temporary suspension of their much-loved music careers causing concerns for current financial stability and future career prospects, mid-career musicians (aged 35–45) suffered more severely than their more seasoned colleagues (53 and above; Cohen and Ginsborg). Particularly their lower motivation to practice and engage in collaborative music-making during lockdown was suggestive of limited psychological resilience. Physical activity may, however, provide some potential for alleviation (Spiro et al.). A further survey and interview study in the USA revealed that live music-making decreased and online music-making increased during lockdown, and that music-creation also became less collaborative and more solitary at first (Fram et al.). However, music creators later restored their collective work approaches and sometimes even prioritized communal aspects over matching musical genres with collaborators. As primary sources for inspiration during this time, creators drew heavily upon nature, nostalgic topics, social issues, and their personal lives.

## Conclusion

This Research Topic of papers reveals the benefits of interdisciplinary collaborative research efforts in times of crisis. The outputs have shown clear evidence and future suggestions for how music can support us through times of duress. Across the COVID-19 pandemic, the world has been far more connected *via* technology than ever before, and the speed and efficacy of digital communication has enabled musical activity and research about it to progress as quickly as the pandemic itself. The wide array of musical engagements captured by the research has highlighted the rich cognitive and physical benefits afforded by musical engagement to bring about crucial social convergence in times of culturally imposed spatial distancing.

## Author Contributions

NH conceived of the structure of the Editorial and produced the figure. All authors listed have made a substantial, direct, and intellectual contribution to the work and approved it for publication.

## Funding

For this work, NH received funding from the European Union's Horizon 2020 research and innovation programme under the Marie Skłodowska-Curie grant agreement No. 754513, Aarhus University Research Foundation, and seed funding grants from Interacting Minds Center (2020-153, 2021-186). JD's research was funded by the Creativity and Wellbeing Hallmark Research Initiative at the University of Melbourne.

## Conflict of Interest

The authors declare that the research was conducted in the absence of any commercial or financial relationships that could be construed as a potential conflict of interest.

## Publisher's Note

All claims expressed in this article are solely those of the authors and do not necessarily represent those of their affiliated organizations, or those of the publisher, the editors and the reviewers. Any product that may be evaluated in this article, or claim that may be made by its manufacturer, is not guaranteed or endorsed by the publisher.

## Footnotes

^1^E.g., Briggs and Tim Minchin's “HouseFyre”, released on 9th April 2020: https://www.youtube.com/watch?v=6nZQRt4BXr8.

^2^With a single outlier–Belfi et al.–whose data were collected prior to the onset of the pandemic.

^3^See https://www.frontiersin.org/research-topics/14089/social-convergence-in-times-of-spatial-distancing-the-role-of-music-during-the-covid-19-pandemic#impact.
